# The Therapeutic Effect of a Multistrain Probiotic on Diarrhea-Predominant Irritable Bowel Syndrome: A Pilot Study

**DOI:** 10.1155/2018/8791916

**Published:** 2018-12-06

**Authors:** Seok-Hoon Lee, Nam-Seok Joo, Kwang-Min Kim, Kyu-Nam Kim

**Affiliations:** Department of Family Practice and Community Health, Ajou University School of Medicine, Suwon, Republic of Korea

## Abstract

**Backgrounds:**

Recent studies suggest that diarrhea-predominant irritable bowel syndrome (IBS) is associated with intestinal bacterial microflora, colonic inflammation, and small intestinal bacterial overgrowth (SIBO). The purpose of this study was to evaluate the effect of a multistrain probiotic intake on these associated factors in patients with diarrhea-predominant IBS.

**Methods:**

The recruited volunteers were adults who were diagnosed with diarrhea-predominant IBS according to the Rome III criteria. After 8 weeks of probiotic ingestion, changes in gastrointestinal symptoms, fecal microbiome, SIBO, and fecal calprotectin were determined.

**Results:**

There was an increase in beneficial bacteria (41.2 ± 16.8% vs. 53.7 ± 15.3%, *P* = 0.018) and a decrease in harmful bacteria (13.0 ± 13.9% vs. 4.7 ± 4.0%, *P* = 0.010) in the microbial stool analysis. The SIBO prevalence also decreased at the end of treatment. However, the average levels of fecal calprotectin showed a decreasing tendency, without reaching statistical significance (364.4 ± 729.1 mg/kg vs. 200.9 ± 347.6 mg/kg, *P* = 0.375).

**Conclusion:**

Treatment with a multistrain probiotic for 8 weeks led to significant increases in beneficial bacteria in the gut as well as the improvement of gastrointestinal symptoms. This study is registered at the Clinical Research Information Service (KCT0002906).

## 1. Introduction

Irritable bowel syndrome (IBS) is a functional disorder of the gastrointestinal tract characterized by abdominal cramps, bloating, constipation and/or diarrhea, and fecal urgency [1]. The pathophysiology of IBS is not well understood; however, many recent studies suggest that altered gut microbiota such as small intestinal bacterial overgrowth (SIBO) and intestinal microinflammation play an important role [2, 3]. Currently, hydrogen/methane breath test and fecal calprotectin (FC) are commonly used to measure SIBO and microinflammation of the colon, respectively [4, 5].

Among the breath tests for SIBO evaluation, lactulose hydrogen breath test (LHBT) is used to evaluate proximal and distal SIBO, despite its low sensitivity and specificity in patients with rapid intestinal transit [6]. FC is an index that reflects microinflammation of the colon [5]. Several studies have reported that gut inflammation is associated with inflammatory bowel disease as well as IBS, especially diarrhea-predominant IBS [7]. Diarrhea-predominant IBS is associated with microinflammation of the colon as well as SIBO. Next-generation sequencing (NGS) is another method for the characterization of gut bacteria and evaluation of intestinal bacterial status.

Previous studies involving diarrhea-predominant IBS investigated the effects of probiotic ingestion on gut and/or fecal microbiota and FC test [8, 9]. However, these studies did not correlate improvement of gastrointestinal symptoms after probiotic ingestion in patients with IBS with changes in intestinal bacterial microflora, SIBO, and/or colonic microinflammation. Therefore, we conducted a pilot study to investigate the effect of probiotic intake on gut microbiota and gastrointestinal symptoms. The changes in SIBO were evaluated by breath test with lactulose, and microscopic inflammation in the colon was evaluated with FC, as well as digestive symptoms in diarrhea-predominant IBS patients. The primary aim was to assess the degree of abdominal symptom relief and changes in fecal microflora in diarrhea-predominant IBS patients after 8 weeks of multistrain probiotic treatment. The secondary aim was to assess the effect of treatment on SIBO and FC.

## 2. Materials and Methods

### 2.1. Study Populations and Design

The current study was a single-arm, open-label, pilot study. The endpoints of the present study were to evaluate the improvement in gastrointestinal symptoms including diarrhea and changes in microbiome after 8 weeks of probiotic intake. The recruited volunteers were all male (19–70 years) who were diagnosed with diarrhea-predominant IBS according to the Rome III criteria [10]. Exclusion criteria were (1) intake of probiotics including Ther-Biotic® Complete; (2) diagnosed gastrointestinal diseases other than diarrhea-predominant IBS, such as constipation-predominant IBS, mixed-type IBS, Crohn's disease, celiac disease, and ulcerative colitis; (3) past history of intestinal surgery such as gastrectomy or cholecystectomy (except appendectomy); (4) history of viral hepatitis, liver cirrhosis, malignancy, chronic kidney disease, congestive heart failure, and thyroid disease; (5) antibiotic treatment during the previous month; and (6) history of travel to parasite-endemic countries.

**Figure 1:**
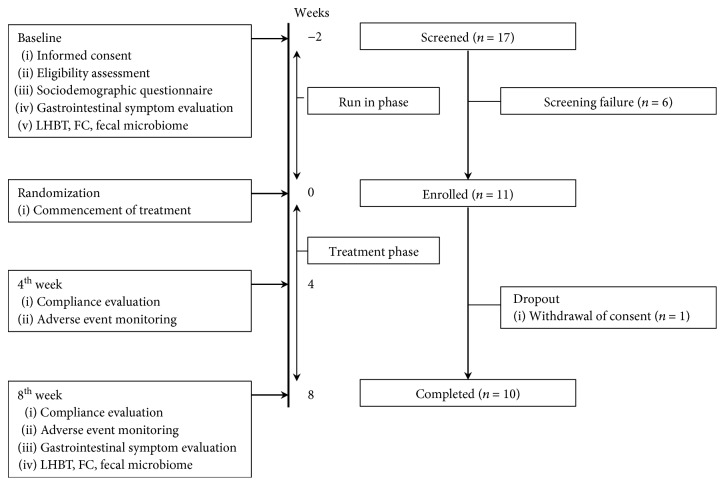
Flow chart of the present study. LHBT: lactulose hydrogen breath test; FC: fecal calprotectin.

During the screening period, 17 subjects were enrolled, 6 were excluded, and 1 patient withdrew consent at the last follow-up, and the final study included 10 participants (Figure 1). Eligible patients underwent gastrointestinal symptom evaluation, LHBT, FC, and fecal microbiome analysis at the baseline. In addition, enrolled subjects were prohibited from taking medications such as antibiotics, prebiotics, prokinetics, and over-the-counter drugs that could affect IBS. After 4 weeks of probiotic ingestion (once daily), gastrointestinal symptoms and adverse events were investigated. Gastrointestinal symptom evaluation, LHBT, FC, and fecal microbiome analysis were conducted at the end of 8 weeks. Safety assessments included recording of the vital signs, physical examination, and laboratory evaluation. The full analysis set (FAS) (*n* = 11) included patients randomized to treatment who received at least one dose of the assigned treatment. The per protocol set (PPS) (*n* = 10) included those who completed the study according to the protocol.

The probiotic agent used in the present study was Ther-Biotic® Complete, a multispecies probiotic combination (Ther-Biotic® Complete; ProThera Inc., USA) designed and marketed for various digestive problems including IBS. Each capsule contains 25 billion active bacteria with 12 different strains: *Lactobacillus rhamnosus* 6.0 billion CFU, *Bifidobacterium bifidum* 5.0 billion CFU, *L. acidophilus* 3.0 billion CFU, *L. casei* 2.5 billion CFU, *L. plantarum* 2.0 billion CFU, *L. salivarius* 2.0 billion CFU, *B. longum* 1.0 billion CFU, *Streptococcus thermophilus* 1.0 billion CFU, *L. bulgaricus* 1.0 billion CFU, *L. paracasei* 0.5 billion CFU, *B. lactis* 0.5 billion CFU, and *B. breve* 0.5 billion CFU.

All patients provided written informed consent. This study was designed, implemented, and reported in accordance with the Korean Good Clinical Practice, with applicable local regulations and with the ethical principles laid down in the Declaration of Helsinki. Study approval was obtained from the institutional review board of Ajou University Hospital (approval no.: AJIRB-MED-FOD-16-252). The clinical trial number was acquired from the Clinical Research Information Service (CRIS registration number: KCT0002906).

## 3. Measurements

### 3.1. Gastrointestinal Symptoms

The patients were scored for severity of abdominal discomfort, dyspepsia, flatulence, and epigastric pain on a ten-point ordinate (numerical rating) scale. Stool form was assessed using the Bristol stool form scale [10]. The Bristol stool form scale is an ordinal scale of stool types ranging from the hardest (type 1) to the softest (type 7) with pictorial representations of each stool type. Types 1 and 2 are considered constipation, while types 6 and 7 are considered diarrhea. Types 3, 4, and 5 are generally considered as the most normal stool forms.

### 3.2. FC and Fecal Microbiology Assay

The fecal samples for FC and fecal microbiology analysis were collected at the beginning and at the end of treatment (8th week). Upon receipt in the laboratory, all stool samples were registered and stored at −20°C. In this condition, the stool samples for FC were sent to the Institute of Applied Technology for Green Cross LabCell (Yongin, Korea) and the stool specimens for microbiology assay were shipped to the ChunLab Inc. (Seoul, Korea) within 48 h after collection, respectively. The level of calprotectin was measured using an ImmunoCAP 250® (Aloka, Japan) with calprotectin FEIA (fluorescence enzyme immunoassay) kit (Phadia AB, Sweden). FC was expressed as mg/kg of feces. The microbiome was analyzed with NGS methods. A total of 10,000 reads were analyzed, with 99% effective sequence reads, suggesting that most of the gut microorganisms were detected. The richness and diversity of samples were determined by Chao1 estimation and Shannon-Weiner diversity index at 3% distance.

### 3.3. LHBT

LHBT was conducted under standard conditions after fasting for 12 h. Before examination, patients used 20 mL of antiseptic mouthwash (0.05% chlorhexidine) to eliminate fermentation by oral bacteria. End-expiratory breath samples were collected immediately before ingestion of 15 mL of syrup containing 10 g lactulose (Duphalac®; Choongwae Pharma Corporation, Seoul, Korea). Samples were obtained every 20 min for 90 min with two collapsible bags using a mouthpiece and a T-valve. The first bag was used to collect dead space air while the second bag was used to collect end-expiratory air. The breath sample was aspirated from the second bag into a 20 mL of a plastic syringe. Hydrogen gas concentration in the sample was measured immediately using BreathTracker SC QuinTron gas chromatography (QuinTron Instrument Company, Milwaukee, WI, USA). A positive LHBT was defined by one of the following criteria: a baseline value of hydrogen gas > 20 parts per million and/or an increase in hydrogen gas above the baseline value of >20 parts per million between 15 and 90 min after lactulose ingestion [10, 11].

### 3.4. Statistical Analysis

All data were presented as the mean ± standard deviation for continuous variables with normal distribution, as median for continuous nonnormally distributed variables, and as the number of patients (percentage) for categorical variables. Each analysis was considered significant at *P* < 0.05 (two-tailed). Comparisons between the groups regarding sociodemographic and baseline clinical variables, FC, and fecal microbial composition were performed using Fisher's exact test or the chi-squared test for categorical variables and the Mann–Whitney test for nonnormally distributed continuous variables. Comparisons between groups regarding the percentage of change in each individual symptom were analyzed using the Mann–Whitney test or *t*-test in accordance with the variable distribution. Comparisons within each group for Bristol stool form scale, FC, and fecal microbial composition were analyzed using Wilcoxon and paired *t*-test. McNemar's or Bowker's test was used for categorical variables. The SAS 9.2 (SAS Institute, Cary, NC, USA) was used for most of the statistical analyses, except the Wilcoxon signed-rank test, which was performed with free open-source software (R3.3.1; R Foundation for Statistical Computing; http://www.r-project.org). The last-observation-carried-forward analysis was used for missing values, except safety set analysis.

## 4. Results

**Figure 2:**
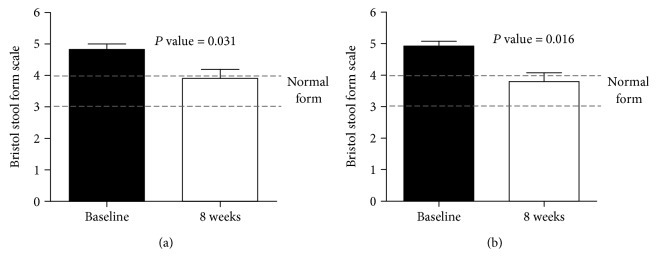
Bristol stool form scale before and after probiotic ingestion in FAS and PPS groups. (a) FAS group and (b) PPS group Bristol stool form scales before and after consumption of probiotics were analyzed using the Wilcoxon signed-rank test. FAS: full analysis set; PPS: per protocol set.

**Figure 3:**
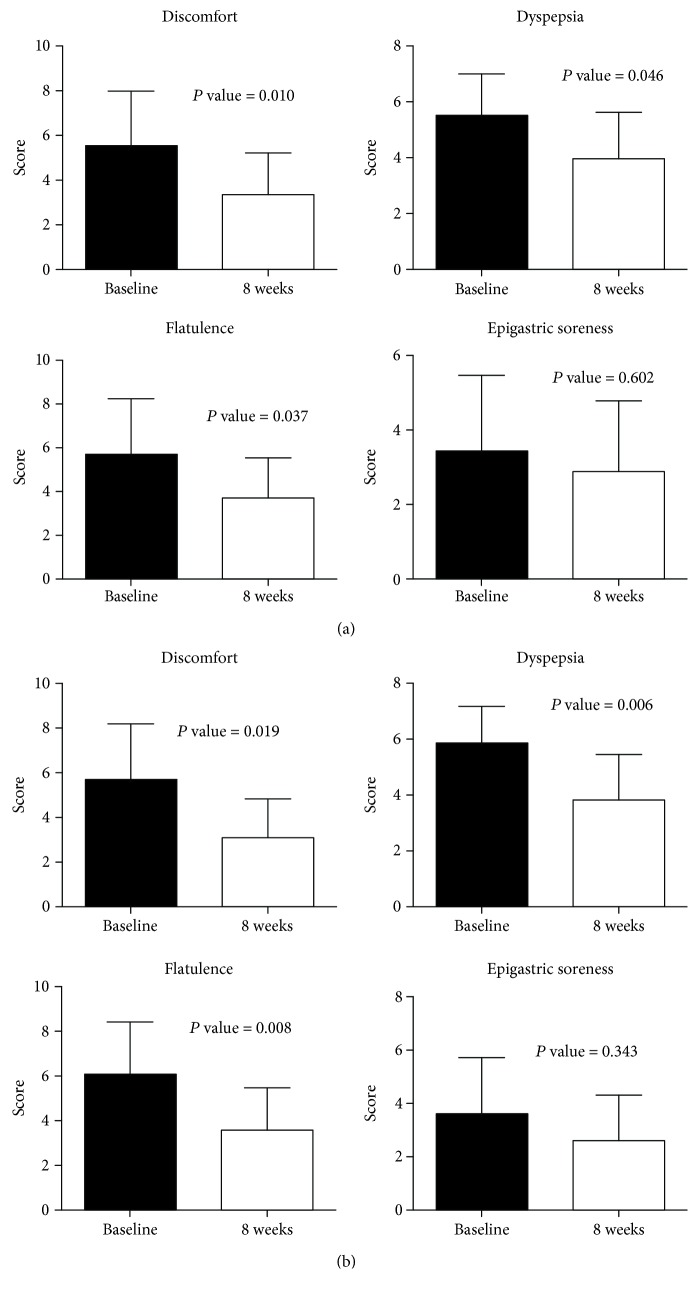
Gastrointestinal symptoms before and after probiotic ingestion in FAS and PPS groups. Gastrointestinal symptom scales before and after consumption of probiotics were analyzed using the Wilcoxon signed-rank test. (a) FAS group. (b) PPS group. FAS: full analysis set; PPS: per protocol set.

Demographic characteristics of patients are listed in Table 1. The mean age, height, and weight of the patients (mean ± standard deviation) were 47.7 ± 10.1 years, 170.1 ± 6.9 cm, and 71.4 ± 6.5 kg, respectively. The mean duration of IBS was 9.6 ± 9.0 years, ranging from 6 months to 30 years. One patient reported prior history of appendectomy carried out more than 30 years ago, and other patients reported the absence of any history of past gastrointestinal surgery. In both FAS and PPS groups, scores on the Bristol stool form scale improved significantly after probiotic treatment (baseline vs. after treatment; 4.8 ± 0.6 vs. 3.9 ± 0.9, *P* = 0.031, 4.9 ± 0.6 vs. 3.8 ± 1.0, *P* = 0.016, respectively) (Figure 2). Abdominal discomfort, dyspepsia, and flatulence were significantly improved in both FAS and PPS groups (FAS group: 5.5 ± 2.4 vs. 3.4 ± 1.9, *P* = 0.010; 5.5 ± 1.5 vs. 4.0 ± 1.7, *P* = 0.046; and 5.7 ± 2.6 vs. 3.7 ± 1.8, *P* = 0.037; PPS group: 5.7 ± 2.5 vs. 3.1 ± 1.7, *P* = 0.019; 5.8 ± 1.3 vs. 3.8 ± 1.6, *P* = 0.006; and 6.1 ± 3.7 vs. 3.6 ± 1.9, *P* = 0.008, respectively) (Figure 3). However, epigastric soreness was not significantly improved in either group (FAS group: 3.5 ± 2.1 vs. 2.9 ± 1.9, *P* = 0.602; PPS group: 3.6 ± 2.1 vs. 2.6 ± 1.7, *P* = 0.343).

In the fecal microbial analysis, operational taxonomic units decreased statistically significantly after 8 weeks in both FAS and PPS groups (FAS group: 251.1 ± 60.7 vs. 198.7 ± 63.0, *P* = 0.018; PPS group: 245.8 ± 61.2 vs. 188.1 ± 55.1, *P* = 0.017) (Table 2). We also analyzed 34 bacterial genera (e.g., *Bifidobacterium* and *Lactobacillus*) associated with health benefits. We investigated 14 genera of harmful bacteria including *Escherichia*, *Clostridium*, and *Haemophilus*. In the FAS group, nine patients showed an increased number of beneficial bacteria (42.9 ± 16.9% vs. 54.3 ± 14.6%, *P* = 0.020) and nine patients demonstrated a decrease in the number of harmful bacteria at the end of treatment compared with baseline (12.5 ± 13.3% vs. 4.9 ± 3.8%, *P* = 0.010). Furthermore, we analyzed 5 genera of lactic acid bacteria (*Bifidobacterium*, *Lactobacillus*, *Lactococcus*, *Leuconostoc*, and *Weissella*). The average number of lactic acid bacteria increased 4.7 times at the end of treatment compared with baseline (0.89 ± 1.0% vs. 4.2 ± 5.1%, *P* = 0.010). In the PPS group, nine patients showed increase in beneficial bacteria (41.2 ± 16.8% vs. 53.7 ± 15.3%, *P* = 0.018) and nine patients demonstrated decreased harmful bacteria (13.0 ± 13.9% vs. 4.7 ± 4.0%, *P* = 0.010). The average number of lactic acid bacteria after treatment increased 4.4-fold (1.03 ± 1.03% vs. 4.56 ± 5.16%, *P* = 0.010).

Six out of 11 patients tested positive for LHBT at baseline, and two out of six patients turned negative after 8 weeks of probiotic treatment (Table 3). In both FAS and PPS groups, the average FC levels were not significantly altered after treatment, although there was a tendency to decrease (FAS group: 323.4 ± 684.9 mg/kg vs. 180.4 ± 327.1 mg/kg, *P* = 0.375; PPS group: 364.4 ± 729.1 mg/kg vs. 200.9 ± 347.6 mg/kg, *P* = 0.375). No patients showed elevated levels of methane in LHBT. No serious adverse events were reported in any patient. Compliance was optimal in all groups.

## 5. Discussion

The aim of this pilot study was to evaluate the clinical effects of multistrain probiotics in diarrhea-predominant IBS and its effect on SIBO and FC. A multistrain probiotic significantly improved the digestive symptoms (abdominal discomfort, dyspepsia, and flatulence) and stool consistency evaluated by Bristol stool form scale. In addition, this study evaluated whether probiotic supplementation altered intestinal microflora. We found that probiotic treatment resulted in an increase in the number of beneficial bacteria and a decrease in the number of harmful bacteria, along with a decrease of SIBO prevalence. Furthermore, although the levels of FC did not reach statistical significance, the overall clinical outcomes pointed to the clinical effectiveness of the probiotic, suggesting its direct relevance to clinical practice.

We used multistrain probiotics including *Lactobacillus* species, *Bifidobacterium* species, and *Streptococcus thermophilus* in this study. Several clinical studies including randomized controlled trials investigated the role of multistrain probiotics in diarrhea-predominant IBS. Placebo-controlled studies of patients using various probiotic mixtures demonstrated significantly greater improvement in stool consistency, alleviated IBS symptoms such as abdominal pain, and satisfaction with bowel habits, quality of life, and fullness compared with the control groups [12, 13]. In particular, a study using multistrain probiotics (*Lactobacillus acidophilus*, *L. plantarum*, *L. rhamnosus*, *Bifidobacterium breve*, *B. lactis*, *B. longum*, and *Streptococcus thermophilus* 1.0 × 10^10^ CFU) very similar to those used in our study showed that the probiotic mixture effectively relieved the overall IBS symptoms and improved stool consistency in diarrhea-predominant IBS patients. However, a recent study showed that an 8-week treatment with BIO-25 comprising three genera (*Lactobacillus*, *Bifidobacterium*, and *Streptococcus*) improved symptoms in women with diarrhea-predominant IBS but was not superior to placebo [8]. Therefore, since data are still conflicting, high-quality clinical studies are necessary to determine the efficacy of multistrain probiotic in diarrhea-predominant IBS patients. Our results did not show a statistically significant improvement in epigastric pain, which may be due to the mild epigastric pain at the baseline. In addition, we evaluated changes in the composition of fecal flora using NGS to determine the role of probiotics in diarrhea-predominant IBS. We observed a decrease in harmful bacteria and an increase in beneficial bacteria, especially the increase of *Bifidobacterium* and *Lactobacillus* species. These results suggest that the ingested multistrain probiotic reached the large intestine and led to improvement of intestinal symptoms. Our results are consistent with several studies into the composition of fecal microflora following the intake of probiotic mixture by Asians [13, 14]. However, operational taxonomic units showed a decrease after the consumption of probiotic mixture. Although a few studies have shown that the consumption of multistrain probiotic results in increased diversity of intestinal bacteria in diarrhea-predominant IBS [13, 14], a recent systematic review of randomized controlled trials reported that probiotic intake did not affect the fecal microbiota composition in terms of diversity or richness compared with placebo in healthy adults [15]. Therefore, this relationship is still controversial and further investigations are needed.

To provide additional insight into the pathophysiology of IBS, we selected SIBO and FC as secondary outcome variables. First, we found that 6 out of 11 patients were SIBO-positive and two out of six were converted to negative after 8 weeks of probiotic exposure. These results are consistent with a recent published systemic review [16], which suggested that probiotic supplementation benefited patients with SIBO. Probiotic therapy was an effective option for SIBO decontamination, reduction in hydrogen concentration, and abdominal pain relief. The cutoff values of FC are 51 (10–59 years of age) and 112 (over 60 years of age), respectively [17]. In the current study, the average of FC (323.4 mg/kg and 364.4 mg/kg for FAS and PPS, respectively) at baseline was higher than the cutoff values. These results suggest that IBS is associated with colonic microinflammation. Despite lack of statistical significance, our data showed that the FC value decreased after probiotic administration. Only one previous study of IBS and probiotics evaluated FC as an inflammatory marker [8]. However, this study demonstrated that the value of FC did not decrease after 8 weeks of probiotic treatment. This discrepancy may be due to the mean value of the initial FC not as high as 20 *μ*g/g in pretreatment subjects and differences in probiotic species and dosages or variations in inclusion criteria of the subjects, sample size, and study design.

The study limitations are as follows. First, our data analysis involved a small number of subjects. Thus, results of our hypothesis-generating study should be confirmed by randomized, double-blind, placebo-controlled trials in a large series of diarrhea-predominant IBS patients. Second, LHBT is associated with the possibility of false positives in patients with fast bowel movements [6]. Thus, the validity and interpretation of the LHBT in SIBO are ongoing controversy. In fact, it has been suggested that LHBT positivity in IBS patients may be related to rapid intestinal transit and not SIBO [18]. However, the North American Consensus has recently demonstrated that not only the breath test using glucose but also LHBT are valuable for the diagnosis of SIBO [19]. Third, our study was coincidentally recruited only men, probably because it was a small pilot study. Therefore, future research needs to be done with designs that include both men and women. Despite these limitations, our study is the first of its kind to demonstrate that multistrain probiotic intake improved gut flora, decreased inflammatory changes in the colon, and resulted in symptomatic improvement of SIBO.

## 6. Conclusion

In summary, the current study has demonstrated that the consumption of multistrain probiotic led to an increase in beneficial bacteria in the gut as well as improvement of gastrointestinal symptoms. This preliminary study analyzed the bacterial composition in intestine, SIBO, and FC after ingestion of multistrain probiotic in a specific and homogeneous group of male patients with diarrhea-predominant IBS. Therefore, large-scale studies are needed to confirm the findings.

## Figures and Tables

**Table 1:** Patient demographics.

FAS group (*n* = 11)	Mean ± SD	Median	Range
Gender (men, %)	11 (100)		
Age (years)	47.7 ± 10.1	46	32–64
Height (cm)	170.1 ± 6.9	170	160.8–184.6
Weight (kg)	71.4 ± 6.5	70.4	58.0–79.0
Duration of IBS (year)	9.6 ± 9.0	8	0.5–30
Past history of GI surgery, no. (%)	1 (9.1)		
Smoking status, no. (%)			
Nonsmoker	2 (18.2)		
Ex-smoker	4 (36.4)		
Current smoker	5 (45.5)		
Alcohol intake, no. (%)			
Never	3 (27.3)		
Previously	2 (18.2)		
Current	6 (54.5)		
Exercise, no. (%)			
Yes	8 (72.7)		
No	3 (27.3)		

FAS: full analysis set; IBS: irritable bowel syndrome; GI: gastrointestinal; SD: standard deviation.

**Table 2:** Fecal microbiome analysis before and after probiotic ingestion.

	Group	Mean ± SD		*P* value	95% CI
		Baseline	8 weeks		
Observed OTUs (ChaO1)	FAS	251.1 ± 60.7	198.7 ± 63.0	0.017	−93.8, −11.2
	PPS	245.8 ± 61.2	188.1 ± 55.1	0.017	−102, −13.3
Beneficial bacteria	FAS	42.9 ± 16.9	54.3 ± 14.6	0.020	2.3, 20.6
	PPS	41.2 ± 16.8	53.7 ± 15.3	0.018	2.7, 22.5
Deleterious bacteria	FAS	12.5 ± 13.3	4.9 ± 3.8	0.010	−19.5, −0.7
	PPS	13.0 ± 13.9	4.7 ± 4.0	0.010	−19.2, −0.8
Lactic acid bacteria	FAS	0.89 ± 1.0	4.2 ± 5.1	0.010	0.3, 8.9
	PPS	1.03 ± 1.0	4.56 ± 5.16	0.010	0.553, 8.9

Beneficial bacteria were defined as genera, which are beneficial to health such as *Bifidobacterium* and *Lactobacillus*. Deleterious bacteria were defined as genera, which are harmful to health such as *Escherichia*, *Clostridium*, and *Haemophilus*. SD: standard deviation; CI: confidence interval; OTUs: operational taxonomic units; FAS: full analysis set; PPS: per protocol set.

**Table:** (a) Lactulose hydrogen breath test

Positive subjects at baseline (%)	Positive subjects at 8th week (%)
6 (54.5)	4 (40)

**Table:** (b) Fecal calprotectin

	Mean value at baseline (mg/kg)	Mean value at 8th week (mg/kg)	*P* value
FAS group	323.4 ± 684.9	180.4 ± 327.1	0.375
PPS group	364.4 ± 729.1	200.9 ± 347.6	0.375

Comparisons of the fecal calprotectin value before and after consumption of probiotics were analyzed using the Wilcoxon signed-rank test. FAS: full analysis set; PPS: per protocol set.

## Data Availability

The data used to support the findings of this study are included within the article.

## Abbreviations

IBS: Irritable bowel syndrome
SIBO: Small intestinal bacterial overgrowth
FC: Fecal calprotectin
LHBT: Lactulose hydrogen breath test
NGS: Next-generation sequencing
FAS: Full analysis set
PPS: Per protocol set.
